# Laparoscopic radical gastrectomy for gastric cancer: an anatomical approach to right mesogastrium excision and its clinical significance

**DOI:** 10.3389/fonc.2025.1573018

**Published:** 2025-04-16

**Authors:** Guofeng Pan, Zhixing Guo, Likui Huang, Weihong Zhang, Suping Li, Jian Chen, Jihuang Wu, Jianbin Weng, Zipeng Zhu, Jianjin Lin, Junpeng Li, Yanchang Xu

**Affiliations:** ^1^ Department of Gastroenterological Surgery Unit 1, The Teaching Hospital of Putian First Hospital, Fujian Medical University, Putian, Fujian, China; ^2^ Department of Gastroenterological Surgery Unit 1, Putian First Hospital, The Affiliated Hospital of Putian University, Putian, Fujian, China; ^3^ Institute of Minimally Invasive Surgery, Putian University, Putian, Fujian, China; ^4^ Department of Operating Room, The Teaching Hospital of Putian First Hospital, Fujian Medical University, Putian, Fujian, China; ^5^ Department of Gastroenterology, The Teaching Hospital of Putian First Hospital, Fujian Medical University, Putian, Fujian, China

**Keywords:** laparoscopic radical gastrectomy, gastric cancer, mesogastric anatomy, right mesogastrium, complete mesogastrium excision

## Abstract

**Objective:**

Radical gastrectomy for gastric cancer involves the en-bloc resection of the primary tumor and complete excision of the mesogastrium. However, the surgical boundaries and techniques for removing lymph nodes above the pylorus during gastric cancer surgery remain unclear. We aimed to investigate a novel, standardized approach for excising the right mesogastrium in gastric cancer patients undergoing suprapyloric lymphadenectomy, focusing on surgical techniques and outcomes.

**Methods:**

Our surgical technique includes identifying three key elements of the mesogastrium: the encircling portion, the suspension point, and the connecting segment. Using these anatomical landmarks, we resect adipose tissue containing lymph nodes from the right mesogastrium and perform root ligation of the right gastric vessels. We then perform D2 lymphadenectomy combined with complete mesogastrium excision (D2+CME). We retrospectively analyzed clinical data from 376 patients who underwent laparoscopic radical gastrectomy with lymph node dissection for gastric cancer, comparing outcomes between laparoscopic suprapyloric lymph node dissection guided by mesogastric anatomy and traditional methods.

**Results:**

A total of 376 patients were included, with 166 undergoing laparoscopic radical gastrectomy with D2+CME and 210 receiving traditional laparoscopic D2 gastrectomy. No significant differences were observed between the groups in age, body mass index, comorbidities, ASA score, tumor differentiation, tumor location, or surgical approach (*P*>0.05). The D2+CME group harvested significantly more lymph nodes than the traditional D2 group (43.84 ± 5.01 vs. 33.18 ± 2.96, *P*<0.001). The number of positive lymph nodes was also higher in the D2+CME group (6.12 ± 0.89 vs. 2.86 ± 0.55, *P*<0.001). The number of lymph nodes harvested from the right mesogastrium was greater in the D2+CME group (3.41 ± 0.48 vs. 1.32 ± 0.37, *P*<0.001). Intraoperative blood loss was lower in the D2+CME group (5.67 ± 0.41 vs. 9.96 ± 0.77, *P*<0.001), and dissection time was shorter (27.22 ± 1.50 vs. 31.31 ± 1.53, *P*<0.001). No significant difference was found in the number of positive lymph nodes in the right mesogastrium (*P*>0.05).

**Conclusion:**

D2+CME is a feasible and effective approach for laparoscopic radical gastrectomy for gastric cancer. The mesogastric anatomical-guided method for suprapyloric lymph node dissection is safe, reliable, and improves lymph node dissection quality while reducing operative time.

## Introduction

Gastric cancer remains a significant global public health issue. In 2020, there were over 1 million new cases of gastric cancer worldwide, with an estimated 769,000 deaths. The incidence ranks fifth, and the mortality ranks fourth globally (1). Early-stage gastric cancer is asymptomatic and difficult to detect, and symptoms related to gastric cancer usually indicate that the disease has progressed. For advanced gastric cancer, D2 radical surgery is still the internationally recognized standard treatment. However, the recurrence rate of advanced gastric cancer is approximately 30-45% (2). After T4a gastric cancer resection, nearly 40% of patients show lymph node metastasis outside the lymphatic tissue in the fat tissue (3). In traditional gastric cancer lymph node dissection, anatomical vascular rupture and mesenteric damage may lead to the spread of cancer cells into the peritoneal cavity, indicating that the surgical technique still requires improvement. In colorectal surgery, total mesorectal excision (TME) and complete mesocolic excision (CME), guided by the concept of mesenteric anatomy, have significantly reduced local recurrence rates and markedly improved long-term survival (4). Shinohara et al. (5) proposed the concept of the fused mesenteric fascia and mesenteric spaces formed by extensive fusion during the development of the greater omentum. Dissecting these mesenteric spaces to excise the greater omentum is analogous to the “holy plane” emphasized by Heald in the concept of total mesorectal excision (TME) in rectal surgery, which involves removing lymph nodes and adipose tissue surrounded by complete fascia. In light of this, D2 lymphadenectomy plus complete mesogastrium excision (D2+CME) has been introduced and research has confirmed that D2+CME significantly improves local recurrence and long-term survival (6). However, there remain issues regarding the standardization, reproducibility, and quality control of the surgical procedure. Although Xie et al. (7), has described the general morphology and structure of the right mesocolon, the specific and practical boundaries and starting and ending points of the right mesocolon during surgical resection have not been detailed. To address this challenge, we propose an anatomical approach to right mesocolon resection in laparoscopic radical gastric cancer surgery and evaluate its safety and feasibility.

## Materials and methods

### Study patients

In this study, we retrospectively analyzed the data of 376 patients with advanced gastric cancer who underwent laparoscopic radical gastrectomy at the Department of Gastrointestinal Surgery, The First Hospital of Putian, Fujian Province, China, between January 2022 and December 2023. One group underwent laparoscopic radical gastrectomy guided by mesenteric anatomy. This group (n=166) underwent gastrectomy with supra-pyloric lymph node dissection (D2) and CME, defined as the D2+CME group. The other group (n=210) underwent conventional D2 laparoscopic radical gastrectomy based on vascular anatomy, and only received traditional supra-pyloric lymph node dissection. This group was designated as the conventional D2 group. All surgeries were performed by experienced surgeons, each having completed over 500 cases of laparoscopic radical gastrectomy. The surgical procedure and anatomical standards followed the Japanese Gastric Cancer Treatment Guidelines (English edition, 6th edition, January 2023) (8), which includes radical gastrectomy and D2 lymphadenectomy with spleen preservation (including lymph node group 10), and the tumor-related features were defined according to the 3rd edition of the Japanese Gastric Cancer Classification (9).

### Patient selection

This study primarily utilizes a retrospective approach to systematically assess the anatomical approach, safety, efficacy, and oncological outcomes of laparoscopic D2 lymph node dissection combined with complete mesogastrium excision (D2+CME) for right mesogastrium resection. The primary evaluation metrics include the time for lymph node dissection above the pylorus, blood loss, number of lymph nodes harvested, and other relevant indicators. The inclusion criteria are as follows: (1) preoperative diagnosis of primary gastric adenocarcinoma confirmed by endoscopy; (2) clinical staging of locally advanced gastric cancer (cT2-4, N-/+, M0); (3) availability of complete video footage for analysis. The exclusion criteria are: (1) history of upper abdominal surgery (excluding laparoscopic cholecystectomy); (2) intraoperative findings suggesting widespread peritoneal implantation, positive exfoliative cytology, or tumor invasion of adjacent organs (T4b); (3) coexisting with other malignant tumors (including lymphoma and stromal tumors); (4) receipt of neoadjuvant therapy.

### Surgical method for lymph node dissection above the pylorus in laparoscopic radical gastrectomy for gastric cancer under membrane anatomy guidance

#### Understanding of the right mesogastrium

In laparoscopic radical gastrectomy for gastric cancer, lymph node dissection in the suprapyloric region—specifically targeting lymph nodes No. 5 and No. 12—necessitates a paradigm shift from the traditional vessel-guided approach to a mesentery-guided strategy. Traditionally, lymph node No. 12a has been defined along the proper hepatic artery, extending to the first porta hepatis, while lymph node No. 5 follows the first branch of the right gastric vessel to the lesser curvature and its proximal main trunk (10). However, recent insights advocate for the excision of the right mesogastrium as a more anatomically and functionally relevant approach. Gong et al. (11) introduced the concept of the proximal segment of the dorsal gastric mesentery and proposed a classification system dividing the gastric mesentery into six distinct regions, including the left gastric mesentery and the right mesogastrium.

**Figure 1:**
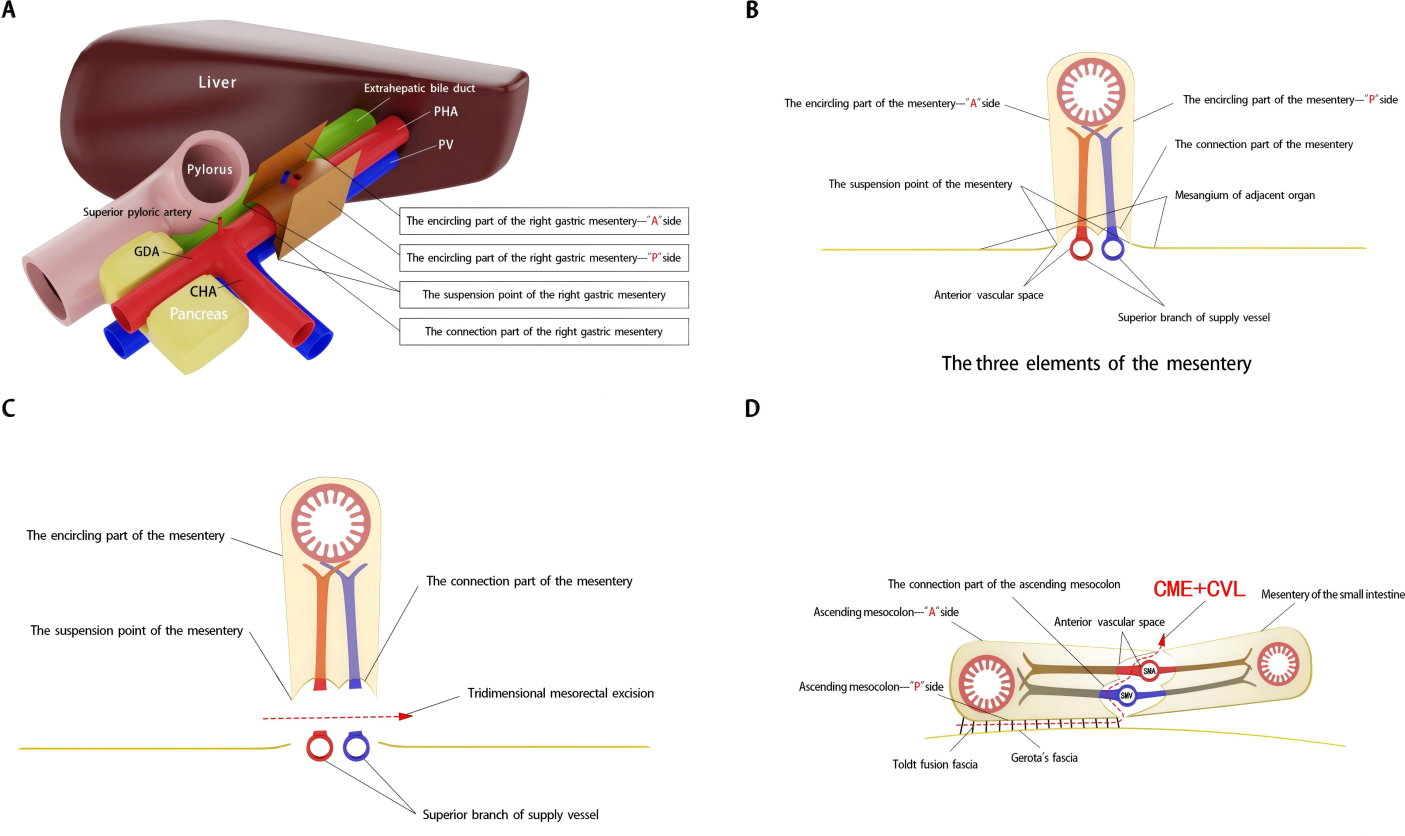
Schematic diagram of right mesogastrium anatomy and three-dimensional mesorectal excision landmarks. **(A, B)** The three elements of the right mesogastrium. **(C, D)** Three-dimensional mesorectal excision illustrating the key anatomical landmarks and surgical approach.

Achieving CME of the right mesocolon requires a precise understanding of its three key components (11) and their accurate identification during surgery. First, the wrapping component consists of the anterior and posterior surfaces of the mesocolon, resembling an envelope, with the anterior surface designated as the A surface and the posterior as the P surface. Due to folding, the mesocolon of adjacent organs forms a fusion fascia with either the A or P surface, which may or may not create a mesenteric bed. Second, the suspension points are located at the lower edges of the anterior and posterior surfaces of the mesocolon, corresponding to the lateral margins of the cross-section of the superior branches of the supplying vessels. Third, the connection component refers to the inferior surface of the mesocolon, where a small, loose, non-vascular space exists between the mesocolon and the superior branch of the supplying vessels. This space, termed the “vascular anterior gap” at this center, serves as a crucial surgical landmark. By performing high-level ligation of the supplying vessels within this gap, CME can be achieved while preserving the three-dimensional integrity of the mesocolon, composed of its anterior, posterior, and inferior surfaces (**Figures 1A, B**). The technical principle of right hemicolectomy with CME, combined with central vascular ligation (CVL), represents a practical application of this anatomical concept (**Figures 1C, D**).

**Figure 2:**
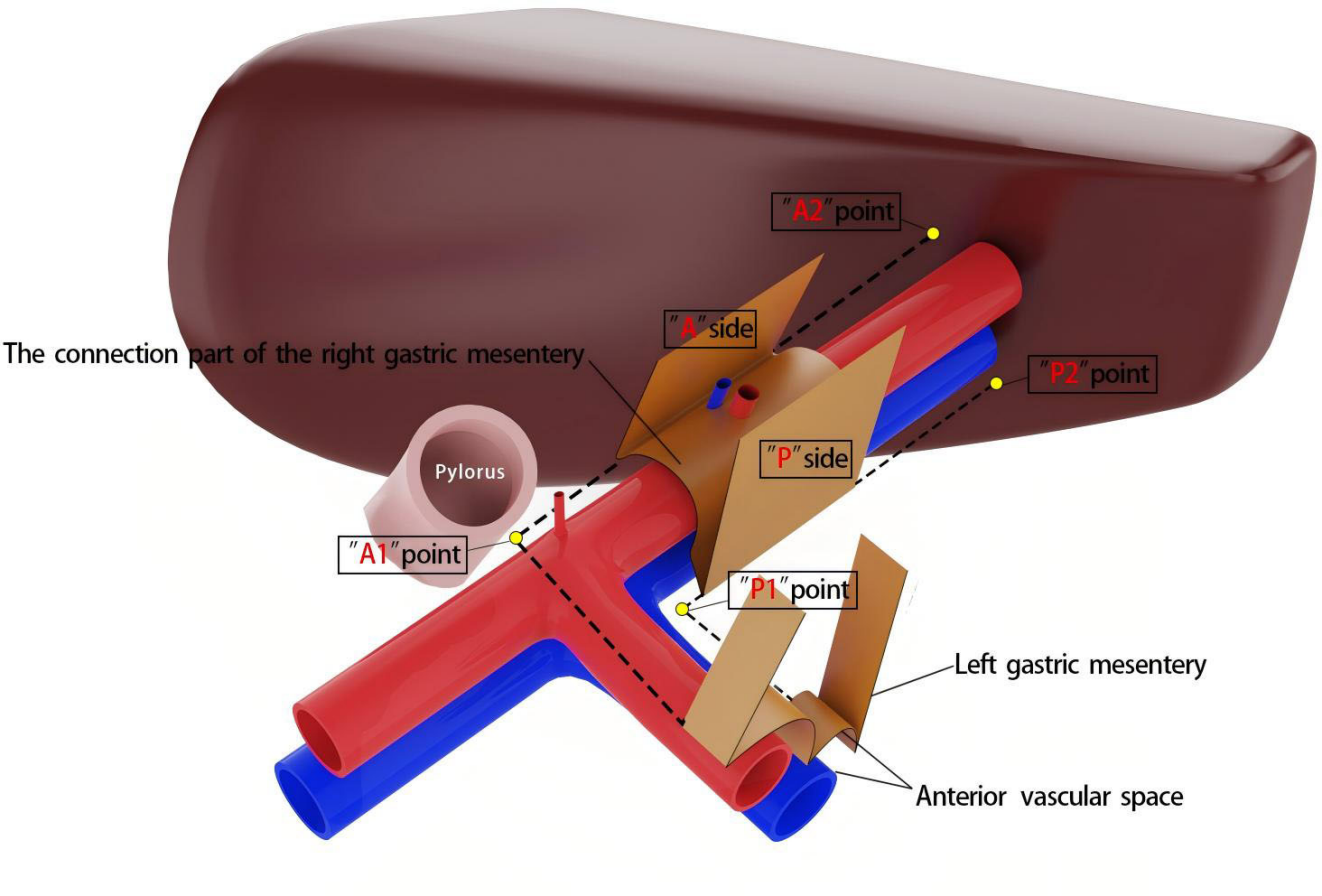
The marking points of the right mesogastrium.

It is important to note that the right mesogastrium straddles the hepatoduodenal ligament. The encircling portion of the right mesogastrium—comprising its anterior (A) and posterior (P) surfaces—does not form a common mesenteric bed with the mesenteries of adjacent dorsal and ventral visceral structures. Consequently, in the schematic diagram, the mesentery covering the dorsal side of the extrahepatic bile duct and the superior branch of the blood-supplying vessels (i.e., the proper hepatic artery and portal vein) is omitted, and only the right mesogastrium is illustrated. To facilitate reader comprehension, the mesentery has been hollowed out and simplified, displaying only its three surfaces for clear identification. The right mesogastrium extends along the superior branch of the blood-supplying vessels, terminating at the first porta hepatis on the cephalic side and continuing leftward as the gastropancreatic fold (referred to as the left gastric mesentery by Gong et al. (12)). The anterior surface (A) of the right mesogastrium converges with the left gastric mesentery at the serosal surface of the gastric wall, posterior to the upper part of the pylorus. This point marks the lower boundary of the anterior surface of the right mesogastrium, designated as point A1, while its terminal attachment at the first porta hepatis is labeled as point A2. Similarly, the posterior surface (P) of the right mesogastrium converges with the left gastric mesentery at the junction of the portal vein and splenic vein, marking the initial suspension point of side P (point P1). The terminal suspension point of side P at the first porta hepatis is labeled as point P2. These anatomical landmarks are illustrated in **Figure 2**. To ensure complete separation of the right mesogastrium from the mesenteries of adjacent organs, we have anatomically marked the starting and ending points of its lower boundary. Notably, the encircling portion of the right mesogastrium (sides A and P) remains entirely free and does not share a mesenteric bed with the adjacent dorsal and ventral organs. Only the connecting segment—the inferior surface of the mesentery—remains attached. Therefore, successful excision of the mesentery necessitates precise entry into this anatomical space and high ligation of the supplying vessels.

#### Specific operation steps

**Figure 3:**
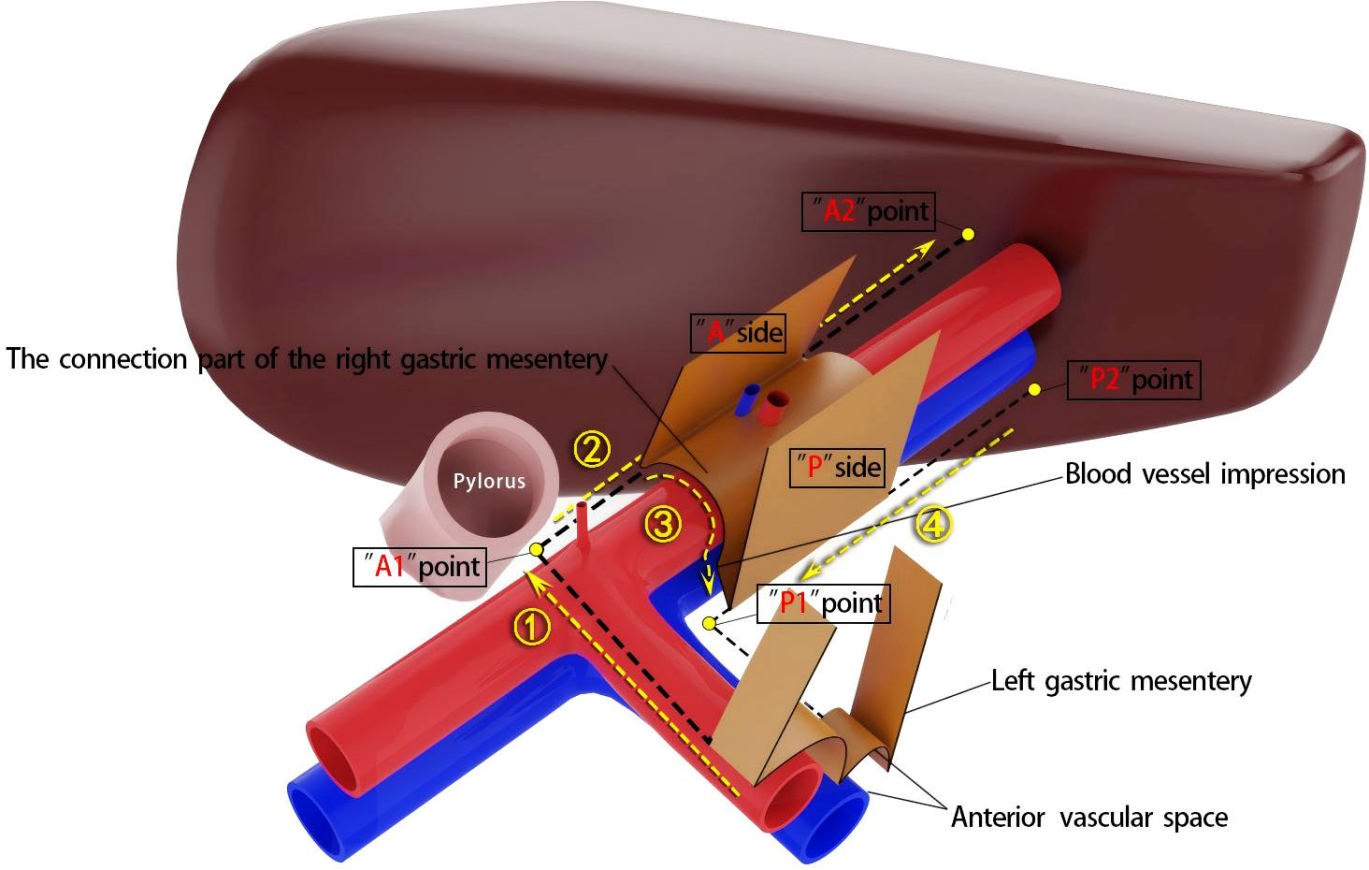
Diagram of steps for the right mesogastrium resection.

**Figure 4:**
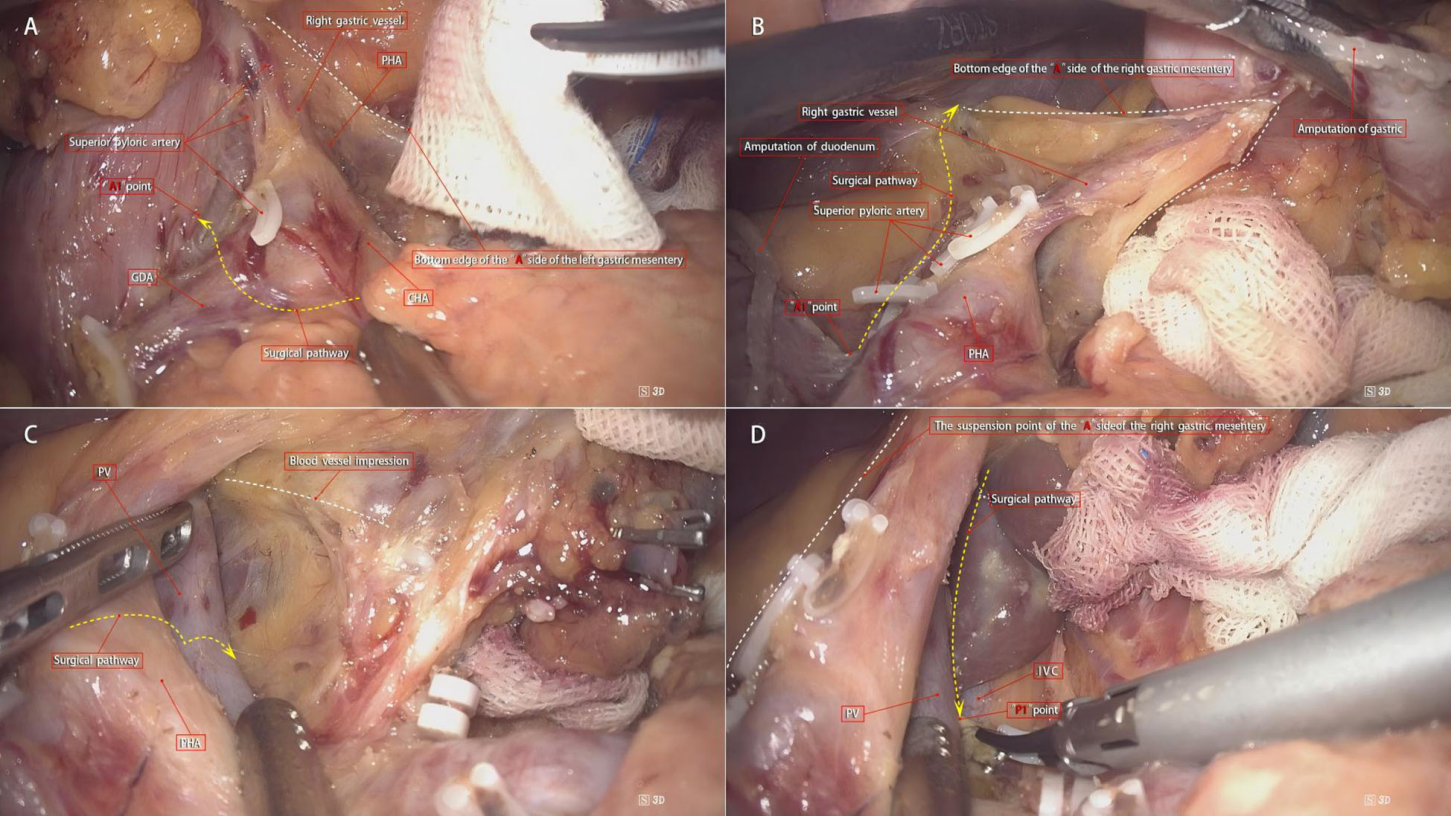
Surgical steps for right mesogastrium dissection. **(A)** Step 1: The right gastric vessel is ligated based on its proximity to the suprapyloric vessel. The triangular structure formed by the common hepatic, proper hepatic, and gastroduodenal arteries is exposed, with side A mesentery (point A1) marked for reference. **(B)** Step 2: The mesentery is incised on the lesser curvature side, and the gastric stump is retracted to expose the right edge of the proper hepatic artery. The quality control standard is complete exposure of the right-edge path of the proper hepatic artery. **(C)** Step 3: The connecting segment of the right mesogastrium is exposed by carefully adjusting the gastric stump. The surgeon uses “pushing” and “cutting” techniques to reveal the left edge of the portal vein, with a vascular imprint visible. The quality control standard is a smooth and complete connecting segment. **(D)** Step 4: The bottom edge of side P of the right mesogastrium is transected at the left edge of the portal vein, completing the excision. The quality control standard is smooth surfaces of the proper hepatic artery and portal vein with clear boundaries.

According to the theory of mesenteric anatomy (7), gastric cancer can be divided into cancer within the gastric mesentery, cancer outside the gastric mesentery, and cancer at the edge of the gastric mesentery. Surgical treatment of gastric cancer can achieve radical results for cancer within the mesentery. Taking laparoscopic distal gastrectomy with D2 radical resection for T3 antral gastric cancer as an example, the surgical method for excising the right mesogastrium is described. A conventional 5-port method is used for the operation, and the excision of the right mesogastrium is completed in 4 steps as shown in **Figure 3**. Step ①: The scene is transferred to the upper edge of the pancreas. The assistant clamps and lifts the entire stomach to maintain appropriate tension, exposing the right side of the upper edge of the pancreas, where the side A suspension point (i.e., the bottom edge) of the left gastric mesentery can be seen. The surgeon makes an incision here and enters the pre-common hepatic artery space, and then extends this space to the right until the pylorus is exposed, revealing the right edge of the proper hepatic artery and the origin of the gastroduodenal artery. During this process, one to several suprapyloric vessels and right gastric vessels can be seen, which are ligated and divided at a high position. It should be noted that if the right gastric vessel is close to the suprapyloric vessel, it can be directly ligated; if it is far from the suprapyloric vessel, it can be dealt with in the next step. The side A mesentery of the right mesogastrium can be seen behind the upper part of the pylorus, which is also the starting point of the suspension point of side A, that is, point A1. A gauze strip is placed here for subsequent operations. The quality control standard for completion is to expose the triangular structure formed by the convergence of the common hepatic artery, the proper hepatic artery, and the gastroduodenal artery, while maintaining the integrity of the connecting segment where the right mesogastrium and the left gastric mesentery are continuous with each other (**Figure 4A**). Step ②: The scene is transferred to the lesser curvature side of the stomach. The guiding gauze strip placed previously under the side A mesentery of the right mesogastrium can be seen. The mesentery is incised here, and the duodenum is transected. The assistant clamps and pulls the gastric stump upward and to the left, ensuring the tension of the side A mesentery. The surgeon can easily and accurately transect the bottom edge of the side A mesentery along the right edge of the proper hepatic artery towards the porta hepatis. The quality control standard for completion is the complete exposure of the entire right-edge path of the proper hepatic artery (**Figure 4B**). Step ③: Continuing on the lesser curvature side of the stomach, because the bottom edge of the side A of the right mesogastrium has been cut, the assistant adjusts the direction of the gastric stump in real-time and subtly. This is similar to a “page-turning” technique, ensuring a clear and accurate exposure of the bottom surface (i.e., the connecting segment) of the right mesogastrium. The surgeon uses a combination of “pushing” and “cutting” dissection methods to naturally move in the pre-vascular space, transitioning from the surface of the proper hepatic artery to the surface of the portal vein and revealing the left edge of the portal vein. Here, the bottom surface (i.e., the connecting segment) of the right mesogastrium can often be seen, and a vascular imprint is formed due to the depression between the superior-level supplying artery and vein. The quality control standard for completion is the smooth and complete connecting segment of the right mesogastrium (**Figure 4C**). Step ④: At the left edge of the portal vein, the suspension point (i.e., the bottom edge) of the side P of the right mesogastrium is transected from the direction of the porta hepatis to the angle between the portal vein and the splenic vein, thus completing the complete excision of the right mesogastrium. The quality control standard for completion is that the surfaces of the proper hepatic artery and the portal vein are smooth and clean, with clear left and right boundaries (**Figure 4D**).

### Traditional surgical method

Dissection of lymph nodes in groups 5 and 12a: The assistant uses the left-hand clamp to lift the antrum of the stomach and the greater omentum upward and the right-hand clamp to push the duodenal bulb outward. The surgeon gently presses the pancreas downward near the bifurcation of the common hepatic artery, making the hepatoduodenal ligament tense, and fully exposes the suprapyloric region from the back. The ultrasonic scalpel is used to dissect the proper hepatic artery along its surface towards the porta hepatis, during which the root of the right gastric artery can be exposed. The assistant gently lifts the right gastric artery upward, and the ultrasonic scalpel carefully dissects it and clips and divides it at the root. Then, continue to dissect to the right and open a “window” on the right side of the dissected anterior leaf of the hepatoduodenal ligament to provide an accurate entry point for the next step of transecting the hepatogastric ligament. The assistant gently lifts the dissected adipose-lymphatic tissue on the surface of the proper hepatic artery upward, and the ultrasonic scalpel continues to dissect upward along its anatomical space until the branches of the left and right hepatic arteries, completely removing the adipose-lymphatic tissue in front of the proper hepatic artery and at the root of the right gastric artery; thus, the dissection of lymph nodes in groups 5 and 12a is completed (13).

### Data collection and follow-up

The clinical data of all patients included in this study were collected and analyzed. The collected data included age, gender, preoperative anesthesia score (ASA), tumor differentiation degree, surgical method, total number of lymph nodes obtained in the right mesogastrium and the number of positive lymph nodes, laparoscopic dissection time of the right mesogastrium, and bleeding volume during the dissection stage. In this study, the TNM staging of tumors was determined according to the staging criteria of the 8th edition of the Union for International Cancer Control (UICC-8) (14). The data of all patients were properly stored to protect patient privacy. This study did not involve ethical issues, and informed consent from patients or their families was not required.

### Statistical analysis

Given the retrospective nature of this study, a formal sample size calculation was not performed *a priori*. Instead, the sample size was determined based on the availability of consecutive patients who met the inclusion criteria during the study period. To assess the adequacy of the sample size, a *post-hoc* power analysis was conducted using the primary outcome of lymph node harvest (mean difference = 10.66, pooled SD = 4.07). Assuming an α level of 0.05 and a β error of 0.20 (power = 80%), the observed sample size (n = 166 vs. 210) provided sufficient statistical power to detect significant differences in lymph node yield (Cohen’s d = 2.62, 95% CI: 2.38–2.86), surpassing the threshold for a large effect size (d > 0.8). These results support the robustness of the findings despite the retrospective study design.

All statistical analyses were performed using R software (version 4.3.0). Continuous variables following a normal distribution were presented as mean ± standard deviation (SD) and compared using an independent-samples *t*-test. For non-normally distributed data, values were expressed as median ± interquartile range (IQR), and comparisons between groups were conducted using the Mann-Whitney *U* test. A *P*-value < 0.05 was considered statistically significant.

## Results

A total of 376 patients were included in this study. Among them, 166 patients (44.15%) were in the D2+CME group, and 210 patients (55.85%) were in the D2 group. There were no significant differences in baseline data between the two groups, including age, body mass index, presence of underlying diseases (including hypertension, diabetes, coronary heart disease, etc.), ASA score, tumor differentiation degree, tumor location, and surgical method (total or distal gastrectomy) (*P*>0.05, **Table 1**).

**Table 1:** Baseline characteristics of participants.

Variable	Total (n=376)	D2+CME (n=166)	D2 (n=210)	*t*/*χ^2^*	*P*
Age, Mean ± SD	64.16 ± 8.96	65.14 ± 8.57	63.38 ± 9.20	1.898	0.058
BMI, Mean ± SD	22.13 ± 2.78	22.16 ± 3.03	22.10 ± 2.56	0.208	0.835
Presence of comorbidities (0=No, 1=Yes), n (%)				0.330	0.566
0	192 (51.06)	82 (49.40)	110 (52.38)		
1	184 (48.94)	84 (50.60)	100 (47.62)		
ASA score (1, 2, 3), n (%)				1.780	0.411
1	8 (2.13)	5 (3.01)	3 (1.43)		
2	257 (68.35)	116 (69.88)	141 (67.14)		
3	111 (29.52)	45 (27.11)	66 (31.43)		
Tumor differentiation (poorly differentiated=1, moderately differentiated=2, well differentiated=3), n (%)				2.237	0.327
1	213 (56.65)	95 (57.23)	118 (56.19)		
2	142 (37.77)	65 (39.16)	77 (36.67)		
3	21 (5.59)	6 (3.61)	15 (7.14)		
Tumor location (1=upper, 2=middle, 3=lower, 4=mixed), n (%)				5.534	0.137
1	182 (48.40)	82 (49.40)	100 (47.62)		
2	82 (21.81)	41 (24.70)	41 (19.52)		
3	71 (18.88)	23 (13.86)	48 (22.86)		
4	41 (10.90)	20 (12.05)	21 (10.00)		
Surgical approach (1=total gastrectomy, 2=distal gastrectomy), n (%)				1.244	0.265
1	284 (75.53)	130 (78.31)	154 (73.33)		
2	92 (24.47)	36 (21.69)	56 (26.67)		

BMI, body mass index; ASA, American society of anesthesiologists.

Tumor Location: Upper third=U (upper) zone of the cardia and gastric fundus, Middle third=M (middle) zone of the gastric body, Lower third=L (lower) zone of the pylorus; Mixed refers to involvement of two or more areas.

In the D2+CME group, the average intraoperative blood loss was 108.31 ml, while in the D2 group, it was 173.81 ml. The difference was statistically significant (*t*=-23.174, *P*<0.001). In the D2+CME group, the average blood loss during the dissection of the right mesogastrium was 5.67 ml, while in the D2 group, it was 9.96 ml. The difference was statistically significant (*t*=-64.869, *P*<0.001). In the D2+CME group, the average total number of lymph nodes dissected was 43.84, while in the D2 group, it was 33.17. The difference was statistically significant (*t*=25.709, *P*<0.001). In the D2+CME group, the average total number of positive lymph nodes was 6.12, while in the D2 group, it was 2.86. The difference was statistically significant (*t*=43.593, *P*<0.001). In the D2+CME group, the average total number of lymph nodes in the right mesogastrium was 3.41, while in the D2 group, it was 1.32. The difference was statistically significant (*t*=47.679, *P*<0.001). Patients in the D2+CME group had a mean of 0.18 positive lymph nodes in the right mesogastrium, compared to 0.16 in the D2 group, demonstrating no statistically significant difference between groups (*t*=2.672; *P*=0.095). In the D2+CME group, the average dissection time of lymph nodes in groups 5 and 12 was 27.22 minutes, while in the D2 group, it was 31.31 minutes. The difference in the average dissection time of lymph nodes in groups 5 and 12 between the two groups was statistically significant (*t*=-25.963, *P*<0.001). See **Table 2** for details.

**Table 2:** Comparison of intraoperative outcomes and lymph node dissection between D2+CME and D2 surgery groups.

Variable	Total (n=376)	D2+CME (n=166)	D2 (n=210)	*t*	*P*
Intraoperative blood loss (ml), Mean ± SD	144.89 ± 30.42	108.31 ± 33.76	173.81 ± 20.63	-23.174	<0.001
Right gastrointestinal mesentery dissection blood loss (ml), Mean ± SD	8.07 ± 2.38	5.67 ± 0.41	9.96 ± 0.77	-64.869	<0.001
Total number of lymph nodes dissected, Mean ± SD	37.88 ± 4.86	43.84 ± 5.01	33.17 ± 2.96	25.709	<0.001
Total number of positive lymph nodes, Mean ± SD	4.30 ± 0.38	6.12 ± 0.89	2.86 ± 0.55	43.593	<0.001
Right gastrointestinal mesentery lymph nodes, Mean ± SD	2.24 ± 0.45	3.41 ± 0.48	1.32 ± 0.37	47.679	<0.001
Right gastrointestinal mesentery positive lymph nodes, Mean ± SD	0.17 ± 0.06	0.18 ± 0.07	0.16 ± 0.05	2.672	0.095
Lymph node dissection time for groups 5 and 12, Mean ± SD	29.51 ± 2.53	27.22 ± 1.50	31.31 ± 1.53	-25.963	<0.001

## Discussion

In the surgical treatment of gastric malignancies, laparoscopic radical resection of the primary lesion with D2 lymph node dissection has become widely adopted. Lymph node dissection remains a crucial and challenging component of gastric cancer surgery. However, even with radical D2 surgery, the recurrence rate remains high (15), and long-term outcomes often fall short of expectations. The limited success of D2 procedures in the diagnosis and treatment of gastric cancer has spurred interest in alternative approaches. Drawing from the successes of total mesorectal excision (TME) for rectal cancer and CME for colonic cancer, there is a growing recognition of the mesentery’s role in cancer spread. Unlike traditional lymph node dissection, which focuses on the removal of regional lymph nodes based on specific stations, mesentery-based surgery emphasizes the complete excision of the mesentery along its natural boundaries. This mesentery-centric approach has been further explored and applied to other digestive tract organs (16, 17). While the fan-shaped mesenteries of the small intestine and colon are well known, the concept of the gastric mesentery is less familiar. This is primarily due to the unique evolutionary development of the stomach and the small, complex nature of its mesentery, which does not follow the typical fan-shaped structure seen in the small intestine and colon. As a result, it has long been assumed that the stomach lacks a mesentery. However, Shinohara et al. (15) explored the embryological development of the gastric mesentery, identified its distinct anatomical features, and highlighted its similarities to the mesenteries of other organs. They proposed the concept of a D2 radical gastrectomy based on mesentery resection, which they termed “systematic gastrectomy”. Although there has been a growing understanding of the gastric mesentery, knowledge of its anatomical structure remains limited. Gong et al. (11, 12) introduced the theory of the proximal segment of the dorsal gastric mesentery using a “table model”. This segment, bounded by major blood vessels such as the common hepatic artery, proper hepatic artery, gastroduodenal artery (GDA), and the main trunk of the splenic artery with its branches, includes six mesenteries, such as the right mesogastrium, which contain corresponding blood vessels and lymphatic vessels (5). Tao et al. (18) conducted an anatomical investigation into the concept of “mesenterization” resection and the “membrane anatomy” concept in radical gastrectomy. They proposed the resection of six mesenteries and introduced eight vascular fascial margins, which include: the right basal fascial margin of the cardia-celiac trunk, the fascial margin between the celiac trunk and the gastroduodenal artery, the fascial margin between the liver and the gastroduodenal artery, the fascial margin of the gastroduodenal artery, the inferior pancreatic fascial margin, the splenic hilum fascial margin, the splenic artery fascial margin, and the left fascial margin of the cardia-celiac trunk. They further suggested that by dissecting and cleaning these eight vascular fascial margins, it would be possible to “mesenterize” and remove tissues such as blood vessels, fat, and lymph nodes within the mesentery. This approach has propelled radical gastrectomy into the era of D2+CME resection, marking a shift from the traditional D2 era.

**Figure 5:**
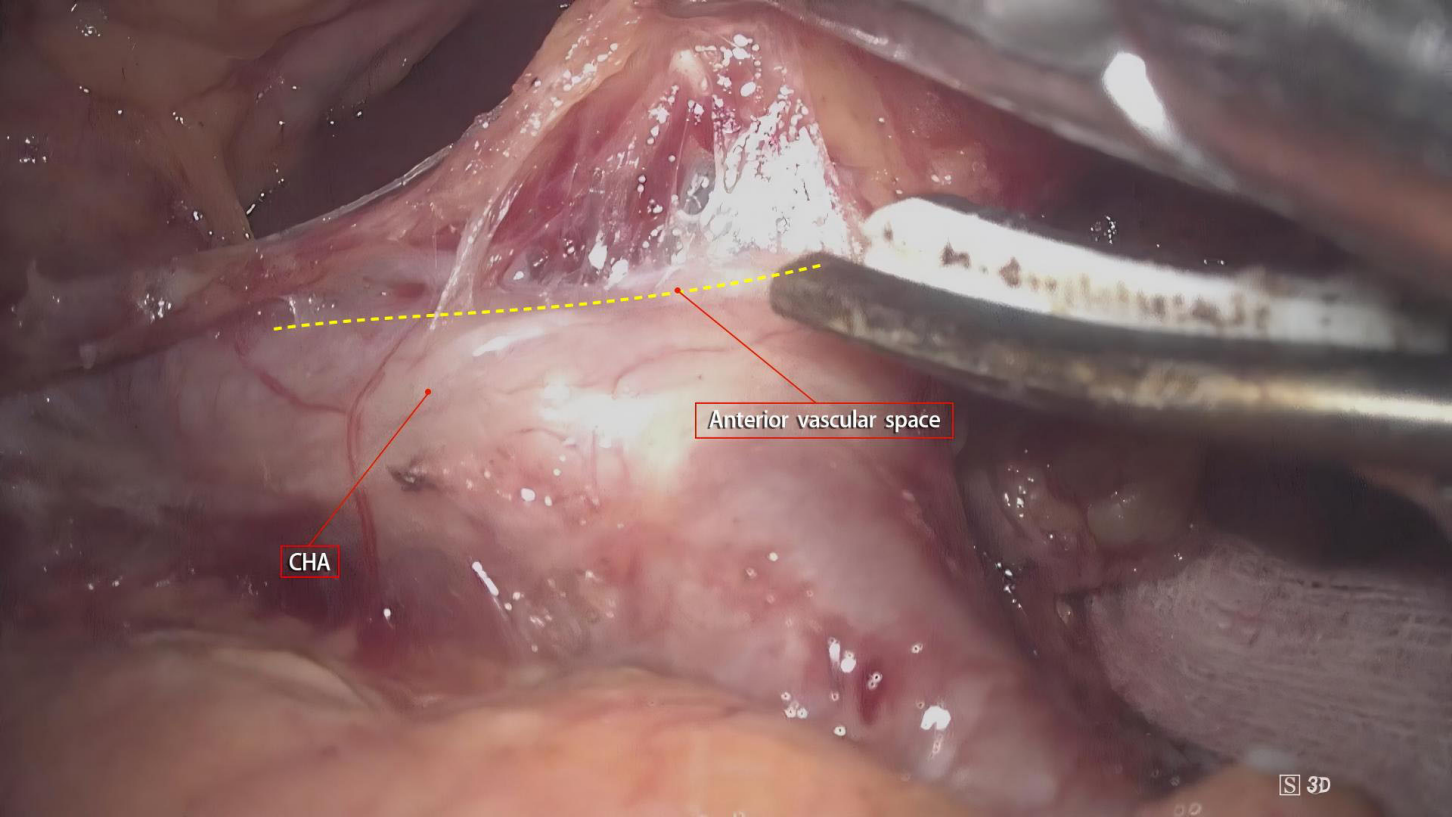
Intraoperative observation of a small, loose, blood-free space between the gastric mesentery and the superior branches of the blood-supplying vessels. This space aids in defining the connection part, addressing the controversy in its current definition.

**Figure 6:**
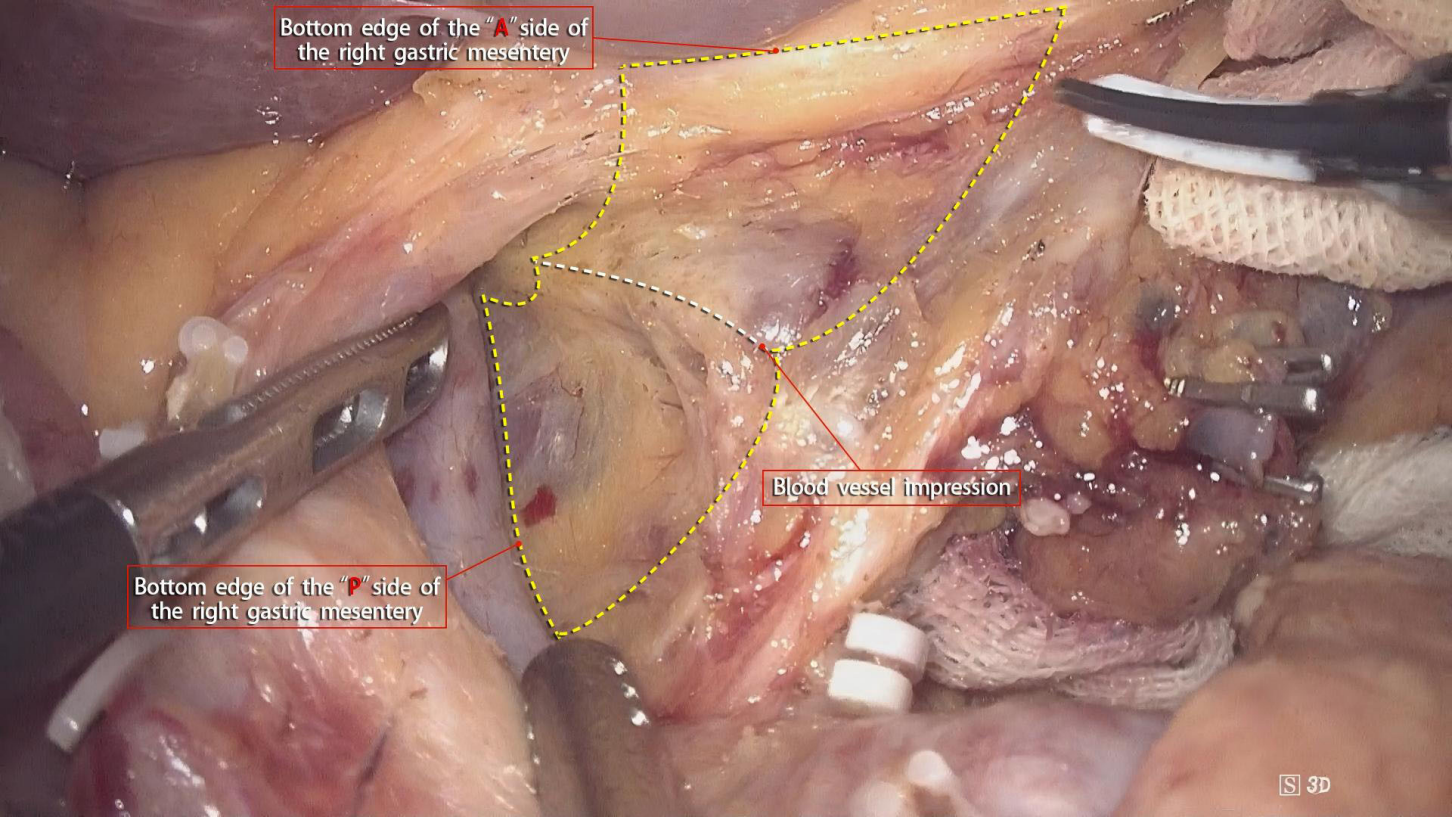
Dissection along the pre-vascular space reveals a smooth, flat mesentery connection with visible blood vessel imprints.

Although gastrointestinal surgery has entered the era of membrane anatomy, the boundaries of its “complete mesentery” have not been fully defined. As a three-dimensional structure, in addition to the proximal and distal resection margins, the mesentery has two sides (front and back) forming an “envelope-like” structure and the bottom surface at the opening. These three surfaces need to be defined. Gong Jianping proposed the concept of the three elements of the mesentery. Personally, I understand that this concept defines the three surfaces of the mesentery accordingly. The understanding of the A and P surfaces is relatively straightforward. After undergoing evolution processes such as rotation and recumbent folding during the embryonic development period, they form fused fascias with or without the mesenteries of adjacent organs as the mesentery bed. The essence of mesentery surgery is to dissociate the fusion. For example, the resection of the right gastroepiploic mesentery is to dissociate the fusion between the A-surface of the mesentery and the colonic mesentery and the fusion between the P-surface of the mesentery and the duodenal mesentery. However, the definition of the bottom surface, that is, the connection part, remains controversial and confusing. In this regard, the author, combined with intraoperative findings, noted that there is a small, loose, blood-free space between the gastric mesentery and the superior branches of the blood-supplying vessels (**Figure 5**). Our center named this space the pre-vascular space. At the same time, Shinohara et al. (15), described and histologically confirmed its existence in an article. By dissecting along this space, the root of the transected gastric mesentery, that is, the mesentery connection part, is smooth and flat, and blood vessel imprints can be seen (**Figure 6**).

Through continuous laparoscopic gastric cancer clinical practice, our center has utilized the clearer anatomical view provided by laparoscopy to identify the morphological structures of the three elements of the gastric mesentery during radical gastrectomy, providing an anatomical theoretical basis for the complete resection of the gastric mesentery. In this study, we applied this idea to the dissection of the supra-pyloric region, defined the dissection boundary of the right mesogastrium, and proposed a reproducible and standardized surgical approach to shorten the learning curve time for beginners of gastric mesentery resection. To accurately achieve the complete resection of the right mesogastrium, the following key points should be followed. In the first step of the operation, the identification and exposure of the suspension points on the A-surface of the right mesogastrium are of great importance. Especially, point A1 is the starting point of the right mesogastrium resection and is related to the quality of the operation. The idea of the exposure process of point A1 (i.e., first dissecting the right side of the upper pancreatic margin) in our center is based on the following considerations: ① The gastropancreatic fold can be well exposed and its tension can be properly maintained by the assistant grasping the left gastric vessels. Thus, the bottom edge of the A-surface of its mesentery can be identified. The “cavitation” effect produced by the main surgeon using an ultrasonic scalpel to cut the bottom edge can easily and correctly enter the pre-vascular space and quickly expand this space, providing the correct guiding direction for the subsequent dissection of the bottom-surface space of the right mesogastrium resection. ② Through the correct dissection of the upper pancreatic margin space, the common hepatic artery and its inferior branch vessels can be well exposed. The spatial connection between the axis of the lower edge of the common hepatic artery and the right edge of the proper hepatic artery determines point A1, avoiding the inaccurate determination of the starting point in the lesser curvature approach and circumventing potential iatrogenic injuries such as biliary tract injuries. ③ Dissecting the right side of the upper pancreatic margin can prioritize the treatment of the supra-pyloric vessels, especially the artery, avoiding iatrogenic injuries caused by the inability to clearly expose the supra-pyloric vessels due to gastric obstruction in the lesser curvature approach. In the second step of the operation, our center routinely transects the duodenum, which brings two benefits: ① After transecting the duodenum, the space becomes larger, the field of view becomes clearer, and there are fewer iatrogenic injuries. ② By pulling the gastric stump by the assistant, the right mesogastrium can obtain good tension, and the bottom-surface space, that is, the pre-vascular space, can be expanded better and faster.

**Figure 7:**
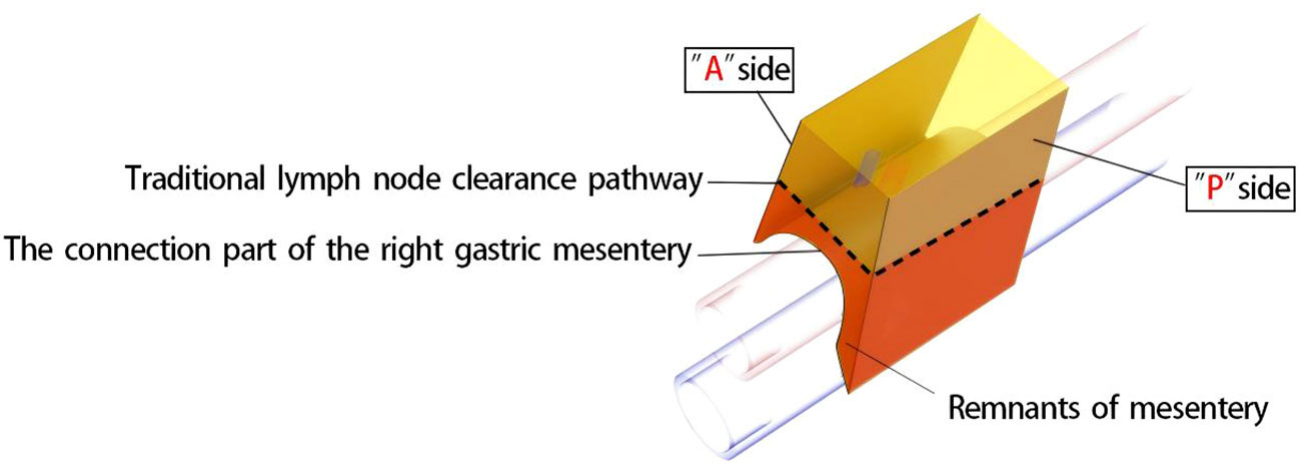
Diagram of the traditional lymph node clearance pathway.

The new right mesogastrium resection method advocated in this article is a self-summary and innovation based on Professor Gong Jianping’s previous surgical methods, aiming to reduce the difficulty of gastric mesentery resection and improve its applicability among young physicians. Professional surgeons often rely on experience and perception to identify the fused fascial space, which poses a great challenge to young physicians. By locating these important anatomical landmarks, it may enable surgeons to more easily apply this new surgical method to achieve the complete resection of the right mesogastrium. Our previous research (19) has confirmed that compared with traditional regional lymph node resection, right mesogastrium resection has the advantages of reducing intraoperative blood loss and increasing the number of lymph nodes dissected while ensuring surgical safety. This is also in line with expectations. As shown in the schematic diagram (**Figure 7**), traditional lymph node dissection surgery can lead to incomplete mesentery resection, damage, bleeding, and incomplete lymph node dissection.

It is important to recognize that anatomical variability among patients significantly influences the feasibility and precision of the procedure. Variations in mesogastrium anatomy, including differences in length, positioning of key vessels, and surrounding tissue structures, can present challenges during surgery. These variations may impact the accessibility and isolation of the right mesogastrium, thus affecting the ease and safety of excision. To address this, preoperative imaging techniques, such as CT and MRI scans, are essential for identifying these anatomical differences. Such imaging enables a tailored approach, allowing the surgical team to plan and adapt the procedure according to the patient’s individual anatomy. Intraoperative techniques, including meticulous dissection and ongoing assessment, further facilitate adaptation to these anatomical variations, ensuring optimal surgical outcomes. Despite the challenges posed by anatomical diversity, our findings suggest that with proper preoperative planning and intraoperative flexibility, the procedure remains feasible for most patients. However, in cases with significant anatomical deviations, additional techniques or modifications to the standard procedure may be required to guarantee patient safety and procedural success.

At the same time, this study has the following limitations. First, this surgical approach was only performed by one professional surgeon. In the future, it is necessary to further verify the reproducibility of this standardized surgical approach among young physicians. Second, the oncological efficacy has not been evaluated. Theoretically, this new surgical approach can not only achieve more thorough dissection of the supra-pyloric lymph nodes but also remove potential cancer cells or cancer nodules in the adipose connective tissue of the right mesogastrium, which is expected to improve long-term survival. Therefore, further research is necessary to determine the oncological benefits of this surgical approach.

In conclusion, this new mesentery-based anatomical method for supra-pyloric lymph node dissection is safe and feasible. It simplifies the complete resection of the right mesogastrium and ensures the quality of the dissection of No.5 and No.12 lymph nodes.

## Data Availability

The original contributions presented in the study are included in the article/supplementary material. Further inquiries can be directed to the corresponding author.
